# Insights into the prognostic potential of total alkaline phosphatase in metastatic castration-resistant prostate cancer treated with radium-223

**DOI:** 10.1590/0100-3984.2023.56.3e2

**Published:** 2023

**Authors:** Gustavo Viani

**Affiliations:** 1 Head of the Radiotherapy Service, Ribeirão Preto Medical School, University of São Paulo (USP), Ribeirão Preto, SP, Brazil. Email: gusviani@gmail.com.

The progress made in the treatment of metastatic castration-resistant prostate cancer (mCRPC) represents a critical landmark in the broader domain of oncology. It manifests in 2-8% of all patients with prostate cancer, creating a clinical scenario that is notoriously challenging to treat^(1)^. Up to 90% of patients with mCRPC develop bone metastasis, which leads to debilitating bone pain in approximately 80% of such patients^(1)^. The advent of radium-223 (Ra-223) therapy as a viable treatment for mCRPC marks a significant turning point in the fight against this aggressive disease^(1,2)^.

Treatment with Ra-223, an alpha emitter, has been successfully employed in treating patients with mCRPC, in whom it has been shown to increase overall survival (OS) and delay skeletal-related events^(1,2)^. Functioning as a calcium analog, Ra-223 specifically binds to osteoblasts, thus inhibiting the growth of bone metastases. It targets cancer cells, induces double-stranded DNA breaks, and alters the metastatic bone microenvironment. In the landmark ALpharadin in SYMptomatic Prostate CAncer (ALSYMPCA) trial, Ra-223 therapy was found to improve OS and delay skeletal-related events in patients with mCRPC and bone metastases, without having a negative impact on quality of life^(1-3)^. In addition, treatment with Ra-223 presents a favorable safety profile, with low myelosuppression rates and minimal gastrointestinal side effects^(3)^. Consequently, treatment with Ra-223 is recognized as an important therapeutic option for patients with mCRPC in whom there are multiple or extensive symptomatic bone metastases and no known visceral metastatic disease^(4)^.

The pursuit of effective prognostic tools in the management of mCRPC remains a pivotal part of ongoing research. Several prognostic factors have been found when using Ra-223 to treat bone metastases in mCRPC. Such factors include the baseline total alkaline phosphatase (ALP) level, hemoglobin level, Eastern Cooperative Oncology Group performance status, and extent of disease on a bone scan^(1)^. The number of Ra-223 cycles administered also affects patient outcomes, studies having demonstrated that five to six cycles of Ra-223 provide OS longer than that achieved with fewer cycles^(2)^. Recent studies have also suggested that early changes in total ALP and in prostate-specific antigen levels are predictive of a treatment response and longer OS^(1,2)^.

Most studies of Ra-223 therapy have been randomized clinical trials, resulting in a lack of real-world evidence studies, especially from Brazil. Real-world evidence studies, which use data from routine practice, offer an invaluable perspective, validating the findings of clinical trials in real-world settings. The aim of the study conducted by Lopes et al.^(5)^, published in the current issue of Radiologia Brasileira, was to fill that void by examining the behavior of total ALP in patients with mCRPC undergoing Ra-223 therapy in a real-world setting in Brazil and evaluating OS in the same population. The authors retrospectively evaluated 97 patients undergoing treatment between February 2017 and September 2020. The patients were stratified according to their total ALP levels at baseline (normal or elevated), with a total ALP response defined as a reduction of 30% or more from baseline at the 12-week mark. For those with elevated baseline total ALP, treatment response was also defined as a reduction of 10% or more in total ALP after the first treatment cycle. The authors defined OS as the period from the first treatment cycle to the date of death. The results of the study were striking: there was a significant reduction in the median total ALP after each treatment cycle (*p* < 0.05 for all). Week-12 total ALP data were available for 71 of the 97 patients, 26 (36.6%) of those 71 showing a response. Among 47 patients presenting elevated baseline total ALP, 19 (40.4%) exhibited a response. It is noteworthy that OS was longer in the patients with normal baseline total ALP, in those demonstrating a response to treatment (≥ 10% reduction), and in those who received 5-6 cycles of therapy (than in those who received fewer cycles). The Lopes et al.^(5)^ study provided insight into the behavior of biomarkers and disease progression in patients with mCRPC who undergo Ra-223 therapy. The authors found that the level of total ALP (a critical marker of bone turnover in prostate cancer) was significantly reduced following each cycle of therapy, except after the first cycle. Those reductions indicate a positive treatment response, given that total ALP is associated with bone metastases in mCRPC. The authors also demonstrated a relationship between the number of Ra-223 therapy cycles and patient survival. Patients who received more than four cycles had significantly longer OS than did those who received four of fewer cycles. That underscores the importance of completing the full course of therapy. Despite the strengths of the Lopes et al.^(5)^ study, certain limitations must be acknowledged. The retrospective nature of the research limits its ability to establish causality. In addition, the real-world setting could involve uncontrolled variables that might have influenced the results. Missing data, a common issue in retrospective studies, also posed a challenge, especially for the total ALP readings. However, despite those caveats, their study provides vital insights into the application of Ra-223 therapy in a real-world context.
